# MiR-760 enhances sensitivity of pancreatic cancer cells to gemcitabine through modulating Integrin β1

**DOI:** 10.1042/BSR20192358

**Published:** 2019-11-19

**Authors:** Dejun Yang, Zunqi Hu, Jiapeng Xu, Yuan Tang, Yu Wang, Qingping Cai, Zhenxin Zhu

**Affiliations:** Department of Gastrointestinal Surgery, Changzheng Hospital, Second Military Medical University, Shanghai 200003, China

**Keywords:** gemcitabine, ITGB1, miR-760, pancreatic cancer (PC)

## Abstract

Pancreatic cancer (PC) is the most lethal tumor type among human diseases, with low survival rate. The investigation of potent molecular mechanisms involved in PC is still obscure owing to its drug resistance. The purpose of the present study is to disclose the underlying mechanism participating in PC progression and drug therapy, reversing the unpromising treatment outcome. In our research, microRNA-760 (miR-760) was first revealed to be lowly expressed in PC cells. And up-regulation of miR-760 could further suppress PC cell proliferation and boost cell apoptosis, as well as improve gemcitabine sensitivity of PC cells through gain-of-function assays. Besides, RNA-binding protein (RBP) MOV10 interacted with and stabilized Integrin β1 (ITGB1). Furtherly, miR-760 was proved to target Moloney leukemia virus 10 (MOV10) mRNA to decrease MOV10 protein expression, thus promoting the destabilization of ITGB1. At last, rescue experiments validated that up-regulation of ITGB1 remedied the miR-760 overexpression-caused inhibition on biological activities and gemcitabine resistance of PC cells. To summarize, the current inspection demonstrated that miR-760 enhances sensitivity of PC cells to gemcitabine through modulating MOV10-stablized ITGB1, highlighting the role of miR-760/MOV10/ITGB1 pathway in the drug therapy for PC patients.

## Introduction

As one of the most usual and aggressive malignancies worldwide [1], pancreatic cancer (PC) has attracted increasing attention of researchers. In spite of the advances in surgery, radiotherapy and chemotherapy (such as Gemcitabine) [2], prognosis of PC patients is still gloomy, with the postoperative 5-year overall survival (OS) rate less than 5% [3,4]. At present, varying genetic and epigenetic regulations are discovered during PC development, hence, the investigation of novel and effective biomarkers for PC therapy is of practical value.

Previous reports have indicated that non-coding RNAs (ncRNAs) exist widely in organisms and exert an indispensable role in affecting multiple cellular activities of tumors, but have restrained protein-coding function, thereby not involving directly in synthesizing proteins [5]. ncRNAs include various forms, such as microRNAs (miRNAs), long ncRNAs (lncRNAs), circular RNAs (circRNAs) [6]. MiRNAs, a cluster of single-stranded RNAs, are emerging as crucial short endogenous RNAs to alter gene expression post-transcriptionally via base-pairing with target mRNAs and thus cellular processes [7]. For example, miR-27a influences the growth and motility of PC cells via targeting Sprouty2 [8]; miR-548an, transcriptionally silenced by HIF1α/HDAC1, inhibits PC tumorigenesis by down-regulating Vimentin expression [9]; miR-219-1-3p negatively regulating mucin MUC4 expression exerts a tumor-suppressive role in PC [10].

MicroRNA-760 (miR-760) has been revealed to passively modulate cell proliferation, apoptosis, invasion and migration in several tumors, and also reduce the drug sensitivity of cancer cells. For instance, miR-760 suppresses non-small cell lung cancer proliferation and metastasis by targeting ROS1 [11]; miR-760 mediates chemoresistance through inhibition of epithelial–mesenchymal transition in breast cancer cells [12]. Nevertheless, the function and mechanistic involvement of miR-760 in PC remain ill-defined.

According to an early study, multidrug resistance (MDR) to chemotherapy is recognized as one of the leading causes of PC-associated deaths [13]. Chemoresistance can weaken the sensitivity of PC cells to chemotherapeutic drugs and therefore result in treatment failure or even death, among which gemcitabine resistance is usually seen during PC therapy [14]. To date, as a result of multifactorial impacts, the mechanism related to PC cells drug sensitivity is still elusive [15]. At this point, it is vitally pressing to probe more valuable regulators affecting the drug resistance of PC cells.

RNA-binding proteins (RBPs) are generally acknowledged as post-transcriptional controllers of particular genes through binding with mRNAs. Moloney leukemia virus 10 (MOV10) was a novel RBP, whose expression was two- to three-times higher in cancer cells than that in normal cells [16]. And MOV10 was found to be an oncogene in tumors [17,18].

In our research, miR-760 was first revealed to be lowly expressed in PC cells, and up-regulation of miR-760 suppressed PC cell proliferation and boosted cell apoptosis, as well as improved gemcitabine sensitivity of PC cells. Besides, MOV10 interacted with and stabilized Integrin β1 (TGB1). Further, miR-760 was proved to target MOV10 and thus destabilize ITGB1. At last, rescue experiments confirmed that ITGB1 up-regulation restored the miR-760 overexpression-induced inhibition on biological activities and gemcitabine resistance of PC cells. In short, our present study elucidated the association between miR-760-mediated mechanism and gemcitabine sensitivity of PC cells.

## Materials and methods

### Cell culture and treatment

Four human PC cell lines (SW1990, AsPC-1, PANC-1 and BxPC-3) and normal human pancreatic ductal epithelial cell line HPDE6c7 were bought from BeNa Culture collection (Beijing, China) and cultured in RPMI 1640 medium (Gibco, Grand Island, NY, U.S.A.). Culture medium contained 10% fetal bovine serum (Invitrogen Life Technologies, Carlsbad, CA) and 1% penicillin–streptomycin (Invitrogen). Cells were allowed to grow in a humidified incubator at 37°C with 5% CO_2_.

SW1990 and BxPC-3 cell lines (3 × 10^4^ cells/well) were planted in 96-well plates and treated with various concentrations (ranging from 0 to 128 μg/ml) of gemcitabine (Selleck, Houston, TX, U.S.A.) for 72 h.

### Cell transfection

For MOV10 or ITGB1 overexpression, the pcDNA3.1 vector (Invitrogen) was cloned with constructed MOV10 or ITGB1 mRNA sequence (pcDNA3.1/MOV10 or pcDNA3.1/ITGB1), and the empty pcDNA3.1 vector was a negative control. As for the down-regulation of MOV10, shRNAs targeting MOV10 (shMOV10) were synthesized and bought from Invitrogen, and miR-760 mimics and NC mimics (Invitrogen) was employed to up-regulate miR-760. These plasmids were individually transfected into cells utilizing Lipofectamine 2000 (Invitrogen) as guided by the provider.

### RNA extraction and qRT-PCR

The total RNA from PC tissues and cell lines were acquired using TRIzol reagent (Invitrogen) following the guidebook for user. RNA was preserved in RNase-free solution at −80°C and reverse transcribed into cDNA using PrimeScript RT Reagent Kit (Takara, Tokyo, Japan). The acquired cDNA was utilized to conduct real-time PCR with SYBR Green Real-time PCR Master Mix (Takara) under the standard system. Finally, the relative gene expressions were analyzed by comparative method (2^−ΔΔ*C*_t_^), with the normalization of GAPDH and U6. MiR-760 primers, 5′-TCAATCCACCAGAGCATGGATAT-3′ (forward) and 5′-CTCTACAGCTATATTGCCAGCCA-3′ (reverse). ITGB1 primers, 5′-GTCCGGTGCCTTTGTCTCTC-3′ (forward) and 5′-CGGCTCCACTAAGCCAACTT-3′ (reverse). MOV10 primers, 5′-CCATGAGGCACATTGTTACG-3′ (forward) and 5′-AAGTGCTTCACCACCTGCTT-3′ (reverse). GAPDH primers, 5′-ACTAGGCGCTCACTGTTCTC-3′ (forward) and 5′-CGCGAAAGGAAAGAAAGCGT-3′ (reverse). U6 primers, 5′-GACGAATACCGGCGTGAGAA-3′ (forward) and 5′-AAATTCTGTTTGCGGTGCGT-3′ (reverse).

### Cell viability assay

Cell Counting Kit-8 (CCK-8) from Sigma–Aldrich (St. Louis, MO, U.S.A.) was employed to analyze PC cells and normal pancreatic cell line HPDE6c7 viability based on the recommendation of supplier. The transfected or treated SW1990 and BxPC-3 cells in 96-well plates (1 × 10^4^ cells per well) were incubated all night and rinsed three times in phosphate buffer saline (PBS). Cells in each well were mixed with 10 μl of CCK-8 solution at 37°C for 2 h. The optical density (OD value) at 450 nm was measured by a microplate reader.

### Drug sensitivity assay

To estimate their chemosensitivity to gemcitabine, cells were plated on to 96-pore plates and dealt with gemcitabine at the indicated different concentrations of 1, 2, 4, 8, 16, 32, 64 and 128 μg/ml for 3 days. After the incubation, count the quantity of viable cells through CCK-8 assay and calculate the half maximal inhibitory concentration (IC_50_) therewith.

### Colony formation assay

The transfected or treated pancreatic cells were collected and placed into six-well plates at 500 cells per well for 2 weeks. Afterward, colonies were fixed in 4% paraformaldehyde for 10 min and dyed with 0.1% Crystal Violet for 10 min. After rinsing twice in PBS, the stained cells were the number of colonies (>50 cells) were counted and recorded. Then, the pictures were taken.

### Caspase-3 activity detection

Cellular activity of caspase-3 was assessed by the use of Caspase-3 assay kit (Abcam, Cambridge, MA, U.S.A.) following the protocol provided by supplier. A total of 5 × 10^6^ transfected or treated PC cells as well as HPDE6c7 cells were cultured in 50 μl of ice-cold Cell Lysis Buffer, followed by separating cell supernatant and analyzing protein concentration. Thereafter, 50 μl of protein were incubated with 50 μl of 2× Reaction Buffer and 5 μl of 4 mM DEVD-p-NA substrate (200 μM final concentration) for 2 h. The absorbance at 405 nm was determined using a microplate reader (Tecan, Switzerland).

### Luciferase reporter assay

The fragments of MOV10 containing the potential miR-760-binding site were enlarged by PCR and cloned into the pmirGLO dual-luciferase miRNA target expression vector, named as MOV10-wild-type (CACS2-WT). And the sequences above were replaced, forming MOV10-mutant (MOV10-Mut) dual-luciferase vector. A total of 1 × 10^4^ tumor cells were plated into each well of 48-pore plate, followed by co-transfection of luciferase reporters (10 ng) and miR-760 mimics (50 nmol/l) or NC mimics utilizing Lipofectamine 2000 (Invitrogen). Two days later, the luciferase activities were evaluated via the Dual-Luciferase Reporter Assay System (Promega, Madison, WI, U.S.A.) together with GloMax 20/20 LUMINOMETER (Promega) as indicated by the providers.

### RNA immunoprecipitation

RNA immunoprecipitation (RIP) assay was performed as expressed before. To put it simply, BxPC and SW1990 cells were resuspended in lysis buffer. For exploring the interplay between MOV10 and ITGB1, cellular lysates were cultivated with Protein G sepharose beads which were conjugated with anti-MOV10 or anti-IgG (Abcam) at 4°C for 4 h. As for the interaction between MOV10 and miR-760, anti-Ago2 or anti-IgG (Abcam) were purchased. Then treat the immunoprecipitates with proteinase K and DNAse I at indoor temperature for 20 min. Co-precipitated RNAs were regained and subsequently subjected to qRT-PCR detection.

### RNA pull-down assay

Total RNA from PC cells was harvested, and then labelled 60 pmol of NC, ITGB1 and ITGB1 antisense with desthiobiotin (IBA, Germany) and linked them to streptavidin magnetic beads (Thermo Fisher) following the supplier’s protocol. Later, total RNA with NC, ITGB1 or ITGB1 were cultured with antisense streptavidin magnetic beads for approximately 2 h with slow rotation. Next, rinsed the beads using buffer and collected them twice, throwing away the supernatant. In the end, the beads were grown with elution buffer for 10 min, and then magnetically separated. The supernatant with the target proteins was saved for Western blotting analysis.

### Western blot

For the extraction of whole proteins, cells were lysed in RIPA (KeyGen Biotech, Nanjing, China). The protein concentration was quantified using a bicinchoninic acid protein assay kit (Pierce, Rockford, IL). Equal quantities of proteins (50 μg) were electrophoresed by 10% sodium dodecyl sulfate/polyacrylamide gel electrophoresis (SDS/PAGE) and transferred on to polyvinylidene fluoride membranes (PVDF; Millipore, Billerica, MA) later. After sealing with 5% nonfat milk at room temperature for 1 h, the membranes were incubated overnight at 4°C with primary antibodies: anti-MOV10 (Abcam) and anti-GAPDH (Protein Tech Group). Signals were visualized through enhanced chemiluminescence reagent (Thermo Fisher Scientific, Waltham, MA).

### Statistical analysis

All experimental data were determined from at least three separate experiments. Data analysis was performed by Student’s *t* test and one-way ANOVA using GraphPad Prism 6.0 (GraphPad, San Diego, CA, U.S.A.) and SPSS 19.0 statistical software (IBM SPSS, Armonk, NY, U.S.A.). Kaplan–Meier method was utilized to analyze OS. The statistical difference was considered significant with a value of *P*<0.05.

## Results

### Overexpression of miR-760 suppressed PC progression

**Figure 1:**
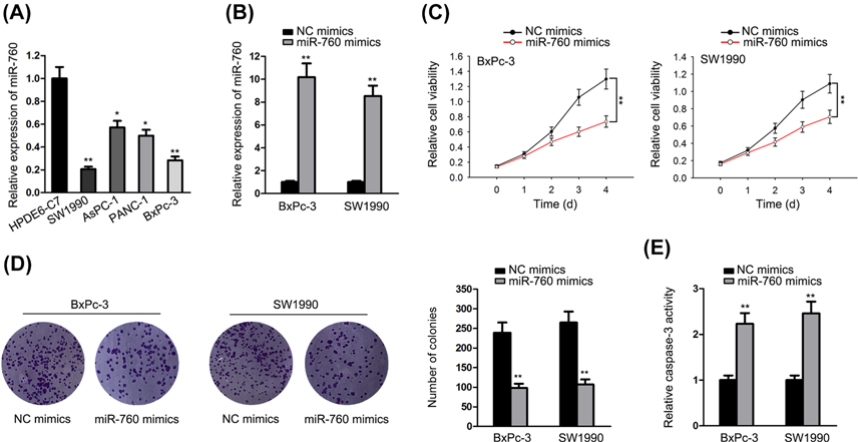
Overexpression of miR-760 suppressed PC progression (**A**) qRT-PCR detection of miR-760 expression in normal pancreatic ductal epithelial HPDE6c7 cells and PC cells (SW1990, AsPC-1, PANC-1 and BxPC-3). (**B**) The overexpression efficiency of miR-760 mimics in BxPC-3 and SW1990 cells was examined through qRT-PCR. (**C,D**) The proliferation ability was analyzed using CCK-8 and colony formation assays. (**E**) The apoptosis ability was dissected using caspase3 activity assay. *P<0.05 and **P<0.01.

To explore the role of miR-760 in PC, we first examined the expression of miR-760 in PC cell lines. The results showed that miR-760 was expressed lower in PC cell lines than in normal cell line HPDE6-C7 (Figure 1A). According to the results of qRT-PCR, miR-760 was expressed lowest in SW1990 and BxPC-3 cells. Thus, we first overexpressed miR-760 in these two cell lines using miRNA mimics (miR-760 mimics). The level of miR-760 was efficiently increased by miR-760 mimics compared with NC mimics (Figure 1B). To identify the biological function of miR-760 in PC, gain-of-function assays were conducted. As illustrated in Figure 1C,D, the cell proliferation was partially inhibited by miR-760 overexpression. Additionally, caspase-3 activity assay revealed that the activity of caspase-3 was increased by miR-760 mimics (Figure 1E), indicating the positive effect of miR-760 on cell apoptosis. The function assays in regard to the cell viability, proliferation and apoptosis in AsPC-1 and PANC-1 cells were also conducted. Reduced cell viability and proliferation as well as promoted cell apoptosis were viewed in miR-760-increased AsPC-1 and PANC-1 cells (Supplementary Figure 1A–D). Further, to explore whether miR-760 functioned in PC specifically or generally. We overexpressed miR-760 expression in normal HPDE6-C7 cells (Supplementary Figure 1E). The effects of miR-760 on the cellular activities of normal cells were experimented. In HPDE6-C7 cells, cell viability, colony formation and cell apoptosis exhibited no change in response to the up-regulation of miR-760, as estimated by CCK-8, colony formation and detection of caspase-3 activity assays (Supplementary Figure 1F–H). Hence, miR-760 functioned in PC cells specifically. In short, overexpressing miR-760 could inhibit cell proliferation and induce cell apoptosis.

### Overexpressing miR-760 sensitized PC cells to gemcitabine

**Figure 2:**
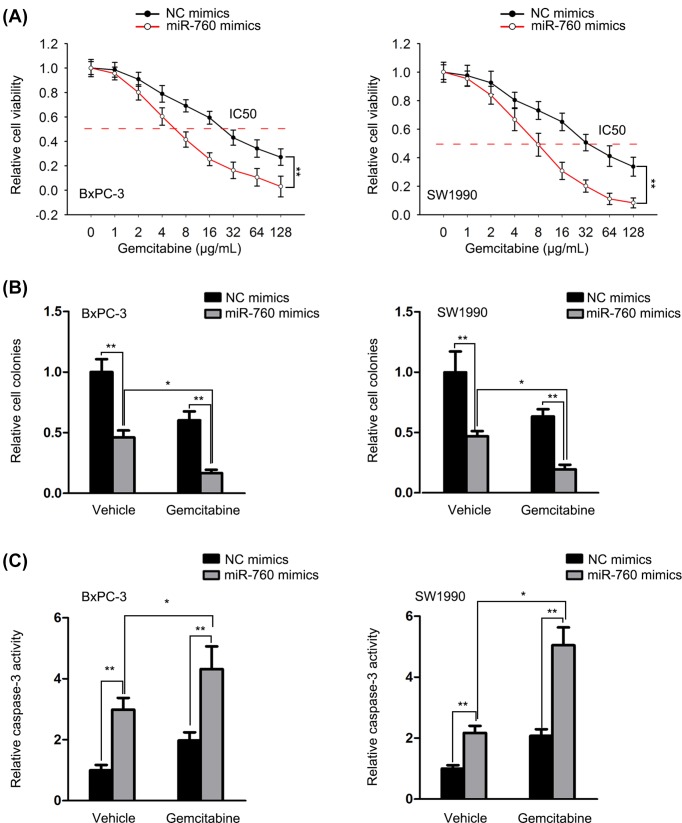
Overexpressing miR-760 sensitized PC cells to gemcitabine Cells were treated with indicated doses of gemcitabine. (**A**) Cell viability was tested through CCK-8 under gemcitabine treatment. (**B,C**) Colony formation and caspase-3 activity results of cell proliferation and apoptosis of PC cells. *P<0.05 and **P<0.01.

Gemcitabine is used as a common chemotherapeutic drug for PC. Here, we further detected the effect of miR-760 on the sensitivity of PC cells to gemcitabine. CCK-8 assay revealed that the IC_50_ value of PC cells to gemcitabine was decreased in response to miR-760 overexpression (Figure 2A). Cells treated with gemcitabine were subjected to functional experiments. The results of colony formation assay showed that gemcitabine-inhibited cell proliferation was further suppressed by miR-760 mimics (Figure 2B). Similarly, the positive effect of miR-760 mimics on cell apoptosis was more efficient in cells treated with gemcitabine (Figure 2C). Taken together, the gemcitabine resistance of PC cells was decreased by miR-760 overexpression.

### Up-regulated ITGB1 in PC interacted with MOV10 and was stabilized by MOV10

**Figure 3:**
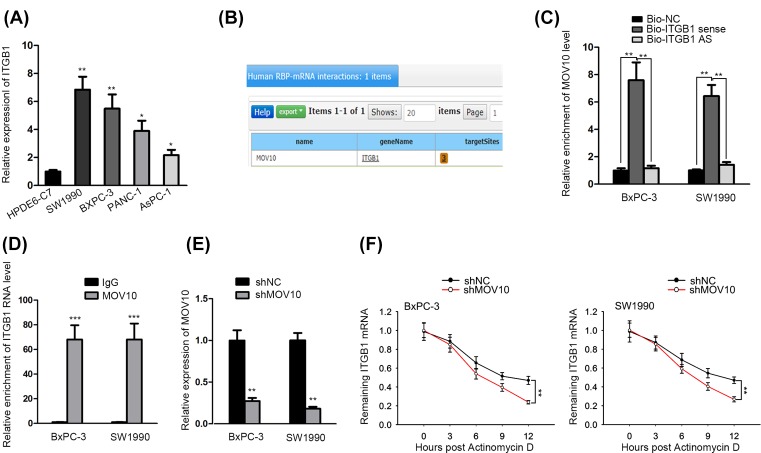
Up-regulated ITGB1 in PC interacted with MOV10 and was stabilized by MOV10 (**A**) qRT-PCR analysis of ITGB1 expression in normal HPDE6c7 cells and PC cells. (**B**) The predicted interplay between MOV10 and ITGB1. (**C,D**) RNA pull-down and RIP assays confirmed the binding of miR-760 to MOV10. (**E**) The knockdown efficacy of shMOV10 was dissected by qRT-PCR. (**F**) ITGB1 mRNA level after actinomycin D treatment was detected using qRT-PCR. *P<0.05, **P<0.01 and ***P<0.001.

The high expression of ITGB1 in PC cells (SW1990, AsPC-1, PANC-1 and BxPC-3) was determined by qRT-PCR (Figure 3A). Gene expression can be regulated from diverse aspects, and the control of RBPs was an increasingly prevalent one. From starBase, a well-known website to predict the interactions between genes, we figured out that MOV10 may bind to ITGB1 mRNA (Figure 3B). RNA pull-down assay illustrated that MOV10 was abundant in the mixture pulled down by bio-ITGB1 sense probe (Figure 3C). As confirmed by RIP assay, ITGB1 was enriched in the compound precipitated by anti-MOV10 (Figure 3D). The MOV10 expression was distinctly elevated in PC cells compared with normal cells (Supplementary Figure S1I). RBPs are recognized to stabilize the mRNA of target genes, hence we prepared shRNAs targeting MOV10 (shMOV10) and actinomycin D for subsequent experiments. MOV10 expression was obviously lowered under shMOV10 treatment (Figure 3E). Finally, qRT-PCR results analyzed that under Actinomycin D treatment, the knockdown of MOV10 dramatically shortened the half-life of ITGB1, hinting that MOV10 might exert its function in PC through stabilizing ITGB1 mRNA (Figure 3F). To sum up, MOV10 could bind to and stabilize ITGB1 mRNA in PC.

### MiR-760 targeted MOV10 to destabilize ITGB1

**Figure 4:**
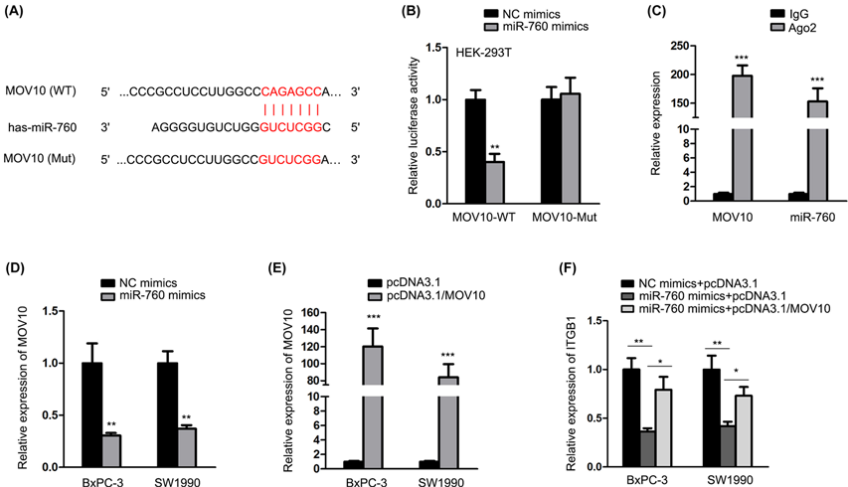
miR-760 targeted MOV10 to destabilize ITGB1 (**A**) The putative binding site between miR-760 and MOV10. The binding sequences and mutant ones were exhibited in the picture. (**B,C**) Luciferase reporter and RIP experiments were implemented to evaluate the interplay of miR-760 with MOV10. (**D,E**) In both the cells, the effect of miR-760 on MOV10 expression and the efficiency of pcDNA3.1/MOV10 treatment were assessed by qRT-PCR. (**F**) qRT-PCR analyzed the regulation of miR-760 and MOV10 on ITGB1. *P<0.05, **P<0.01 and ***P<0.001.

Interestingly, we found from TargetScan website that MOV10 was targeted by miR-760, as presented in Figure 4A. In luciferase reporter assay, the luciferase activity of MOV10-WT was decreased by miR-760 mimics, but that of MOV10-Mut did not change (Figure 4B). In RIP assay, MOV10 and miR-760 accounted for a large proportion of the cocktail of Ago2, respectively (Figure 4C). Also, MOV10 levels were notably declined under miR-760 overexpression (Figure 4D). To deeply affirm the mechanism existing in PC, we examined the influence of miR-760 and MOV10 on ITGB1 expression. MOV10 was up-regulated after the transfection of pcDNA3.1/MOV10 (Figure 4E). qRT-PCR detection determined that ITGB1 was lessened by miR-760 mimics but restored by pcDNA3.1/MOV10 (Figure 4F). Briefly, miR-760 targeted MOV10 so as to destabilize ITGB1.

### Up-regulation of ITGB1 remedied the miR-760 overexpression-caused inhibition on biological activities and gemcitabine resistance of PC cells

**Figure 5:**
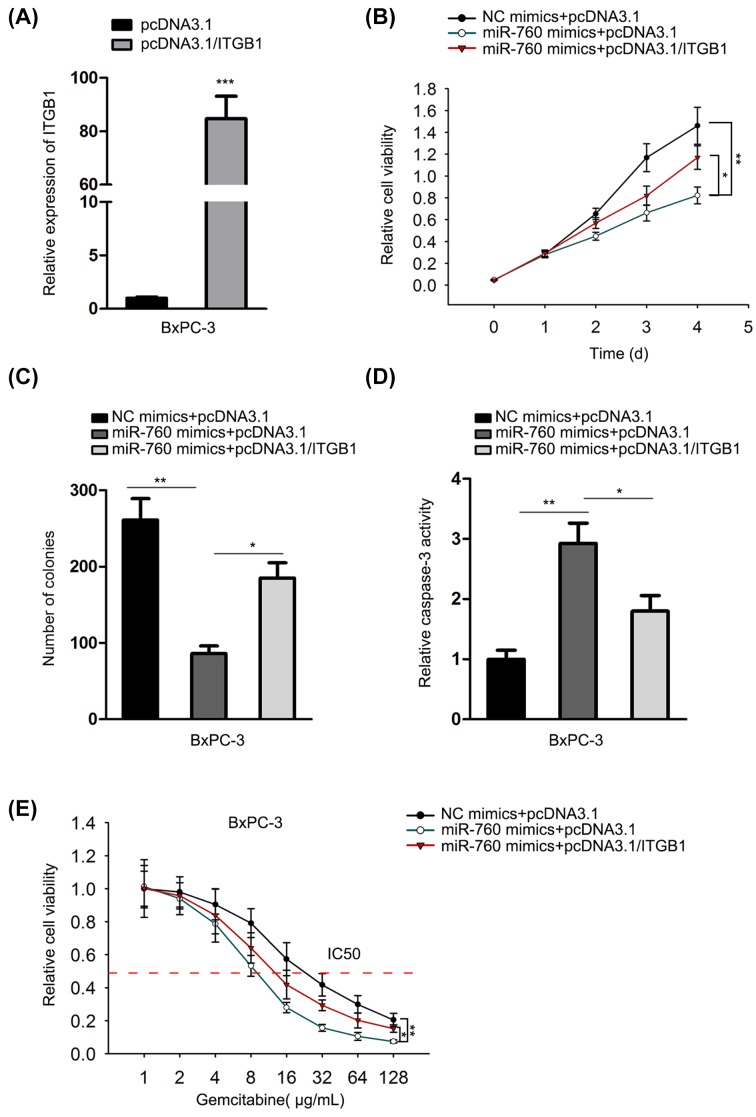
Up-regulation of ITGB1 remedied the miR-760 overexpression-caused inhibition on biological activities and gemcitabine resistance of PC cells BxPC-3 and SW1990 cells were separately co-transfected with NC mimics + pcDNA3.1, miR-760 mimics + pcDNA3.1 and miR-760 mimics + pcDNA3.1/ITGB1 for follow-up assays. (**A**) ITGB1 expression after the transfection of pcDNA3.1/ITGB1 was tested through qRT-PCR. (**B**-**D**) Cell proliferation and apoptosis abilities were dissected by CCK-8, colony formation and caspase-3 activity experiments in transfected PC cells. (**E**) Cells were treated with different doses of gemcitabine to test the gemcitabine resistance of PC cells using CCK-8 experiment. *P<0.05, **P<0.01 and ***P<0.001.

To validate the impact of the regulation mechanism underlying miR-760 on biological processes of PC, we carried out rescue assays. First of all, the transfection efficacy of pcDNA3.1/ITGB1 was confirmed by qRT-PCR (Figure 5A). Cell viability of BxPC-3 cells was repressed by miR-760 mimics but partly recovered by pcDNA3.1/ITGB1 as tested by CCK-8 experiment (Figure 5B). Cell proliferation of BxPC-3 cells was inhibited by miR-760 up-regulation whereas partly rescued by ITGB1 overexpression (Figure 5C). Besides, overexpressing miR-760 stimulated cell apoptosis, which could be partially abrogated by up-regulating ITGB1 (Figure 5D). Moreover, the miR-760 overexpression-reduced IC_50_ value of gemcitabine was regained through co-transfection of ITGB1 up-regulation (Figure 5E). In summary, miR-760 regulated the biological activities and gemcitabine resistance of PC cells via MOV10-stabilized ITGB1.

## Discussion

It has been early declared that miRNAs contribute to tumorigenesis and tumor process owing to their post-transcriptional regulation of target genes. Of note, increasing evidence has demonstrated that miR-760 exerts tumor provoking effect on quantities of carcinomas. For example, miR-760 boosts TRAIL sensitivity in non-small cell lung cancer through reducing the protein FOXA1 [19]; miR-760 suppresses human colorectal cancer growth by targeting BATF3/AP-1/cyclinD1 signaling [20]. These data encouraged us to explore the character of miR-760 in PC progression. Consistently, we found out that miR-760 was significantly reduced in PC cells and up-regulation of miR-760 obviously impaired the proliferation, migration and invasion property, and provoked the apoptosis property of PC.

Gemcitabine (2′,2′-difluoro-2′-deoxycytidine, GEM), is a cytidine analog which prohibits DNA synthesis and also ribonucleotide reductase, serving as a typically first-line regimen for treating pancreatic ductal adenocarcinoma [21]. Nevertheless, the therapeutic outcome of GEM is limited because of inherent and acquired drug resistance [22]. According to a previous research, miR-760 develops a drug-resistant function via impairing doxorubicin resistance in hepatocellular carcinoma by controlling Notch1/Hes1-PTEN/Akt signaling pathway [23]. We suspected that miR-760 participated in the GEM resistance of PC cells. Explorers observed that miR-760 sensitized PC cells to GEM through mechanistic experiments.

The above findings provided impetus for researchers to investigate the feasible mechanism underlying miR-760 in the GEM sensitivity of PC cells. Then, integrin subunit β 1 (ITGB1) was chosen due to its carcinogenic function as accepted in past works. For example, microRNA-3653 targeting ITGB1 restrains the growth and metastasis in hepatocellular carcinoma [24]; microRNA-183-5p inhibits cervical cancer aggressiveness through targeting ITGB1 [25]; lncRNA TUG1/miR-29c axis influences cell proliferation, invasion, and migration in PC by regulating ITGB1 [26]. RBPs are universally known to modulate gene expression. Among the obtained genes, we selected MOV10 as it was revealed to be carcinogenic [16–18]. Our study testified that ITGB1 mRNA was stabilized by MOV10.

Interestingly, MOV10 was predicted to be one target of miR-760 through scanning TargetScan website, which laid the foundation for the follow-up exploration of molecular mechanism underlying miR-760. The present paper indicated that miR-760 regulated the biological activities and gemcitabine resistance of PC cells via MOV10-stabilized ITGB1. Finally, we conducted rescue experiments to affirm the negative effect of miR-760/ MOV10/ITGB1 axis on GEM resistance, revealing that up-regulation of ITGB1 prevents miR-760 overexpression-enhanced GEM sensitivity of PC cells.

All of these investigations presented that miR-760 inhibits cell proliferation and improves GEM sensitivity of PC cells through decreasing MOV10-stablized ITGB1. Therefore, miR-760 symbolizes an underlying target for raising chemotherapeutic efficacy for PC patients.

## Supplementary Material

Supplementary Figure S1

## Abbreviations

Ago2: Argonaute RISC catalytic component 2
AP-1: transcription factor AP-1
BATF3: basic leucine zipper ATF-like transcription factor 3
CCK-8: cell counting kit-8
DEVD-p-NA: Asp-Glu-Val-Asp p-nitroanilide
FOXA1: forkhead box A1
GAPDH: glyceraldehyde-3-phosphate dehydrogenase
GEM: Gemcitabine (2′,2′-difluoro-2′-deoxycytidine)
HDAC1: histone deacetylase 1
HIF1α: hypoxia inducible factor 1 subunit alpha
IC_50_: half maximal inhibitory concentration
ITGB1: integrin β1
lncRNA: long non-coding RNA
miR-760: microRNA-760
miRNA: microRNA
MOV10: Moloney leukemia virus 10
MUC4: Mucin-4
ncRNA: non-coding RNA
OS: overall survival
PBS: phosphate buffer saline
PC: pancreatic cancer
PTEN: phosphatase and tensin homolog
qRT-PCR: quantitative real-time polymerase chain reaction
RBP: RNA-binding protein
ROS1: ROS Proto-Oncogene 1
TRAIL: TNF-related apoptosis-inducing ligand
TUG1: taurine up-regulated 1
