# Supplementary material for: MiR-760 enhances sensitivity of pancreatic cancer cells to gemcitabine through modulating Integrin β1
Source: Biosci Rep. 2019 Nov 19;39(11):BSR20192358. doi: 10.1042/BSR20192358 (PMC6863763; doi:10.1042/BSR20192358)
Supplement: Supplementary Figure S1 [file BSR-2019-2358_supp.pdf]

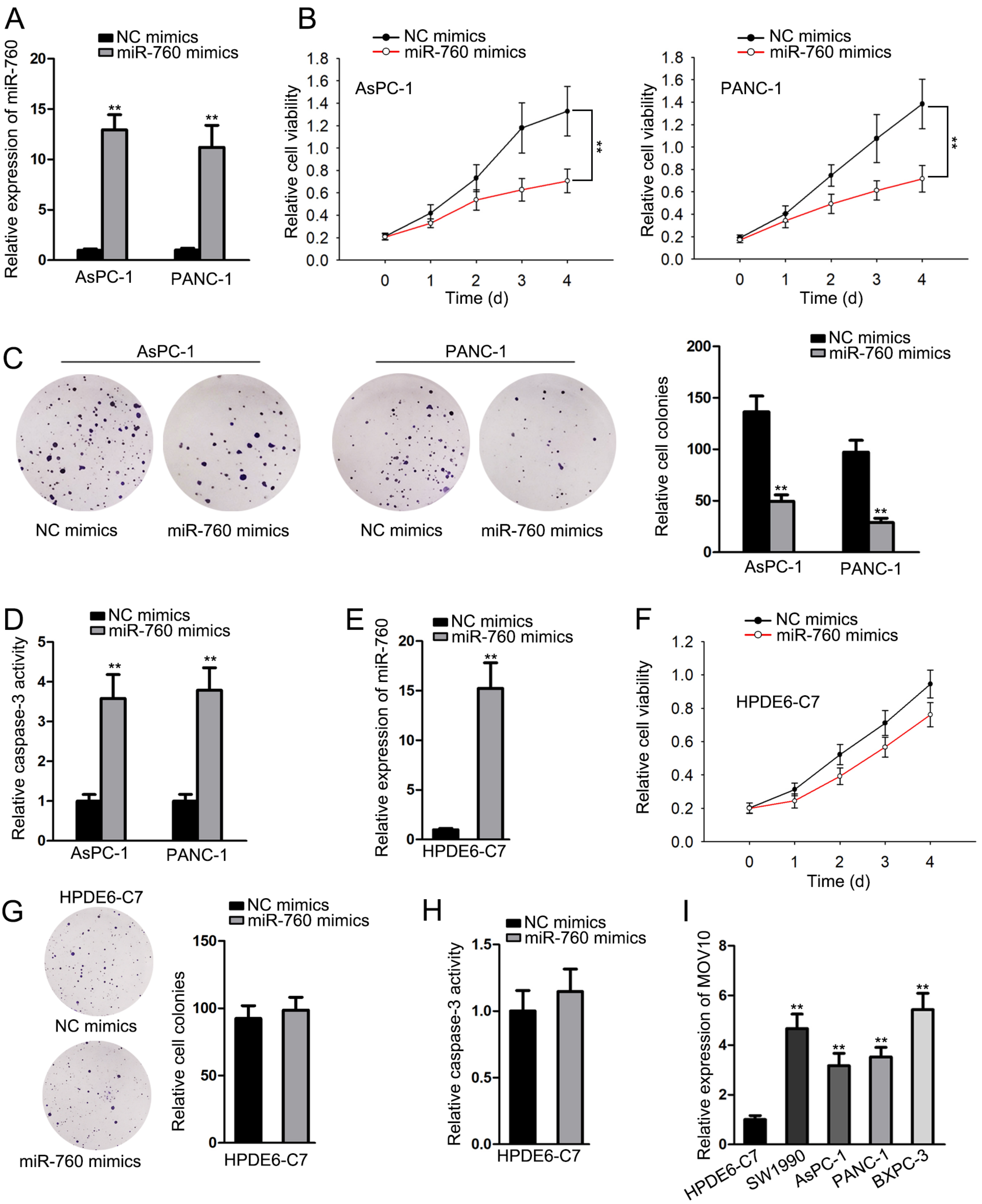
A
NC mimics
miR-760 mimics
Relative expression of miR-760
AsPC-1
PANC-1
B
AsPC-1
PANC-1
Relative cell viability
Time (d)
C
NC mimics
miR-760 mimics
Relative cell colonies
D
Relative caspase-3 activity
E
HPDE6-C7
F
G
H
I
Relative expression of MOV10
SW1990
BXPC-3

Supplementary Figure 1 Function assays in other three pancreatic cells and the analysis of MOV10 expression in all pancreatic cells.

(A-D) The effects of miR-760 overexpression on cell viability, proliferation and apoptosis in AsPC-1 and PANC-1 cells. (E-H) No distinct changes of the cell viability, proliferation and apoptosis existed in HPDE6-C7 cells treated with miR-760 mimics, compared with control cells. (I) qRT-PCR detection of MOV10 expression in normal HPDE6-C7 cells and four PC cells. ** $P < 0.01$.
